# Digit ratio (2D:4D) and maternal testosterone-to-estradiol ratio measured in early pregnancy

**DOI:** 10.1038/s41598-022-17247-3

**Published:** 2022-08-09

**Authors:** Gareth Richards, Ezra Aydin, Alex Tsompanidis, Eglė Padaigaitė, Topun Austin, Carrie Allison, Rosemary Holt, Simon Baron-Cohen

**Affiliations:** 1 School of Psychology, Faculty of Medical Sciences, Newcastle University, 4.32 Dame Margaret Barbour Building, Wallace Street, Newcastle upon Tyne, UK; 2 Department of Psychology, University of Cambridge, Cambridge, UK; 3 Department of Psychiatry, Columbia University Irving Medical Center, New York, NY USA; 4 Autism Research Centre, Department of Psychiatry, University of Cambridge, Cambridge, UK; 5 Wolfson Centre for Young People’s Mental Health, Cardiff University, Cardiff, UK; 6 The Rosie Hospital, Cambridge University Hospitals Foundation Trust, Cambridge, UK; 7 NIHR Cambridge Biomedical Research Centre, Cambridge, UK

**Keywords:** Developmental biology, Endocrinology, Evolutionary developmental biology

## Abstract

The ratio of index to ring finger (2D:4D) has been hypothesised to indicate prenatal androgen exposure, yet evidence for its validity is lacking. We report the first pre-registered study to investigate mothers’ early pregnancy sex hormone concentrations in relation to their children’s digit ratios measured at 18–22-month follow-up. Although the testosterone (T) to estradiol (E) ratio correlated negatively with right hand digit ratio (R2D:4D) and directional asymmetry (right-minus-left) in digit ratio (D_[R−L]_), neither effect remained statistically significant once demographic and obstetric covariates were controlled for. Nevertheless, the multivariate level of analysis did reveal that T correlated positively with left hand digit ratio (L2D:4D) and negatively with D_[R−L]_. However, the first of these effects is in the opposite direction to that predicted by theory. Taken together, the results of our study suggest research with larger samples is required to determine whether digit ratios are valid proxies for maternal sex hormone exposure.

## Introduction

The ratio of second (index) to fourth (ring) finger length (digit ratio or 2D:4D) has been hypothesised to reflect individual differences in prenatal exposure to sex hormones. More specifically, it has been suggested that a high concentration of testosterone^1,2^ or high ratio of testosterone-to-estradiol (T:E)^3–5^ during prenatal development results in low 2D:4D. Researchers typically measure digit ratios for the right hand (R2D:4D) and/or left hand (L2D:4D), though sometimes also examine directional asymmetry (D_[R−L]_). Low R2D:4D relative to L2D:4D has been suggested to reflect high prenatal androgen exposure^6–8^. Small-to-medium sized sex differences (male < female) are reliably observed for R2D:4D (*d* = 0.457) and L2D:4D (*d* = 0.376)^9^, but that for D_[R−L]_ appears to be much smaller (*d* = 0.065)^8^. However, it should be noted that this effect size estimate for D_[R−L]_ may be attenuated; this is due to it having been derived from self-measured finger lengths, which are known to be associated with high levels of random measurement error^8^. Despite enduring popularity, results from studies that have tried to validate digit ratio measures have been equivocal^10,11^. As there is a vast and rapidly growing literature examining 2D:4D in relation to an extensive range of variables across multiple research fields (e.g., psychiatry^12^, social science^13,14^, cancer research^15^, criminology^16^, sports science^17,18^), it is important to consider this lack of consistent evidence rather than rely on the assumption that 2D:4D is a valid and reliable proxy for the prenatal hormonal environment.

Some studies have explored the 2D:4D validity question by examining samples of individuals with medical conditions that affect endocrinological pathways. Complete androgen insensitivity syndrome (CAIS) is an X-linked recessive condition characterised by defective or absent androgen receptors. Despite the presence of normal (or even elevated) androgen levels, this condition results in testosterone being unable to exert physiological effects on the developing tissues. A female-typical phenotype therefore develops in presence of a male-typical (46XY) karyotype and prenatal hormonal environment. Two studies^19,20^ have reported feminised digit ratios in women with CAIS. However, both relied on small samples, and, notably, the variance in 2D:4D did not differ between 46XX and 46XY women. If differential prenatal exposure to the physiological effects of testosterone influences the development of 2D:4D, lower variance should be expected in the latter group than the former^21^. Researchers have also tested for associations between 2D:4D and variations in the trinucleotide CAG repeat sequence located on exon 1 of the androgen receptor gene, a genetic polymorphism believed to influence individual differences in androgen sensitivity^22^. However, although an early small-scale study^23^ reported that low frequencies of CAG repeats, indicative of high sensitivity to androgens, were associated with low (male-typical) R2D:4D and D_[R−L]_, subsequent meta-analyses have not confirmed a meaningful association^24–26^.

Studies of 2D:4D have also been conducted in relation to congenital adrenal hyperplasia (CAH), a suite of conditions characterised by elevated prenatal androgen exposure. A recent meta-analysis detected significant effects for R2D:4D in males and L2D:4D in females (i.e., lower ratios in people with CAH compared to controls), but not for R2D:4D in females or L2D:4D in males^10^. Notably, the effect sizes observed were ~ 50% smaller than those reported in a meta-analysis published a decade previously^9^, implying that early studies may have overestimated the magnitude of these effects. Two studies have also reported feminised 2D:4D in 47XXY men with Klinefelter syndrome^27,28^. However, these findings are difficult to interpret considering that testosterone concentrations measured from amniotic fluid sampled at 16–20 weeks’ gestation did not differ between males with and without this condition^29^.

Evidence of the validity (or lack thereof) for 2D:4D derived from studies of complex medical conditions, such as CAIS, CAH, and Klinefleter syndrome, may be questioned because such conditions affect a wide range of developmental processes. This makes isolating effects attributable to prenatal sex hormones challenging, leading some researchers to examine 2D:4D in more generalisable populations. Twin studies indicate moderate-to-high heritability for 2D:4D, with additive genetic factors explaining most of the inter-individual variation^30–33^. The Twin Testosterone Transfer (TTT) hypothesis (see Ahrenfeldt et al.^34^) has also been examined. This predicts that females with male cotwins will exhibit lower (i.e., more male-typical) 2D:4D ratios than females with female cotwins due to elevated testosterone exposure associated with gestating in close proximity to a male. Although two small-scale studies^30,35^ initially provided some confirmatory evidence, others, including one with a much larger sample size, did not^36–38^. Medland and Loehlin^32^ further reported that 2D:4D ratios were no more similar within dizygotic (DZ) twin pairs than they were within DZ twin/non-twin sibling dyads. This is also inconsistent with a TTT effect on 2D:4D because DZ twins, who, on average, share the same amount of genes identical by descent as non-twin full siblings, would be expected to show elevated concordance for 2D:4D due to their shared prenatal hormonal environment. However, it should be noted that the pattern of results obtained from TTT studies in humans is generally inconsistent^34^, suggesting that if there is an effect for 2D:4D it is likely to be small in magnitude.

Another approach taken in exploring the 2D:4D validity question is to examine digit ratios in relation to actual hormone concentrations obtained during prenatal development. Lutchmaya et al.^3^ reported a negative correlation between the T:E ratio in second trimester amniotic fluid and R2D:4D measured at 2-year follow-up. However, they did not observe a comparable effect for L2D:4D (and did not examine D_[R−L]_). Ventura et al.^39^ later reported a significant negative correlation between amniotic testosterone and L2D:4D (but not R2D:4D) in female (but not male) neonates. However, a reanalysis of the data of Ventura et al.^39^ showed no correlation between amniotic testosterone and D_[R−L]_^40^. Importantly, the statistically significant findings of the studies by Lutchmaya et al.^3^ and Ventura et al.^39^ could not be replicated in an independent cohort^41^.

Some researchers have examined sex hormones measured from the maternal circulation during pregnancy. However, maternal serum testosterone levels assayed during the second and third trimesters has been reported not to correlate with testosterone in second trimester amniotic fluid or umbilial cord blood at birth^42^. Likewise, another study observed no significant correlations between second trimester testosterone concentrations measured from maternal plasma, fetal plasma, and amniotic fluid^43^. However, positive correlations have been reported for estradiol measured from second and third trimester maternal serum and second trimester amniotic fluid^42^. Despite the equivocal evidence for meaningful associations between maternal and fetal sex hormone concentrations, those measured from the maternal circulation have been observed to correlate with phenotypic outcomes in the mothers’ offspring. For instance, elevated maternal testosterone was reported to predict male-typical gender role behaviour in daughters^44^ (though see^45^) and maternal estradiol was reported to correlate positively with autistic traits in sons^46^. There is also evidence of a weak negative correlation between a mother’s second trimester plasma testosterone concentrations and the 2D:4D of her child^39,47^, although this effect has not been observed by all studies^48^. It should also be noted that, although there may be limited transfer of testosterone from mother to fetus via the placenta, testosterone levels are moderately heritable^49^, meaning that correlations between maternal testosterone and outcomes observed in their offspring could reflect genetic rather than hormonal effects^44,45^.

In addition to hormones measured from amniotic fluid and maternal circulation, some researchers have examined concentrations present in umbilical cord blood assayed at birth. Taken together, the findings of these studies indicate no association with 2D:4D^48,50–55^. This is consistent with hormones sampled in this way representing late gestation^56^, whereas sexual dimorphism in 2D:4D has been detected much earlier^57,58^. Considering this, a possible reason for the overall inconsistency in findings from studies attempting to validate digit ratios by correlating them with circulating hormone levels is that such studies have typically assayed those hormones during the second or third trimesters, whereas the critical period for 2D:4D development may exist towards the end of the first trimester^59^.

As it is not possible to measure hormones from the fetal circulation for research purposes, the current study investigates whether maternal sex hormones assayed during late first trimester/early second trimester correlate with the digit ratios of her child. Barrett et al.^60^ recently reported that neither T nor E measured from first trimester maternal serum correlated with infants’ digit ratios (n = 154 males; n = 167 females). However, they did not examine the T:E ratio. Although not numerically or physiologically independent of the individual hormonal measurements, Lutchmaya et al.^3^ notably reported that the T:E ratio in amniotic fluid was a significant predictor of R2D:4D even though T and E themselves were not.

We pre-registered our analysis plan on the Open Science Framework (https://osf.io/bmp6h) and predicted that neither maternal T nor E assayed from late first trimester/early second trimester maternal circulation would be a significant predictor of digit ratio when measured in offspring at 18–22-month follow-up. However, we also predicted that there would be negative correlations between the T:E ratio and R2D:4D and L2D:4D (but not D_[R−L]_). The reason we predicted no effect for D_[R−L]_ is that evidence for the validity of this measure is particularly weak. Although a small study (n = 26 mother–offspring dyads) reported that maternal urinary testosterone-to-estrone conjugate levels in pregnant Titi monkeys correlated negatively with D_[R−L]_ in their offspring, the effect disappeared after covariates were controlled for^61^. Human studies have reported that D_[R−L]_ does not differ between people with and without CAH^10^, and that it does not correlate with sex hormones measured from amniotic fluid^40,41^, umbilical cord blood^48,50^ and second trimester maternal plasma^40^.

## Results

Maternal hormone data (T and/or E) were present for n = 122 (56.22%), digit ratio (direct and/or photocopy) for n = 95 (43.78%), and both for n = 56 (25.81%) (n = 31 females, n = 25 males). For this subsample, mean maternal age at scan was 33.36 years (*SD* = 4.05), mean birth weight was 3406.06 g (*SD* = 503.95), and mean age at follow-up (adjusted for gestational age) was 455.94 days (*SD* = 97.35). Only n = 2 (3.57%) of these mothers reported polycystic ovary syndrome (PCOS), and most experienced hirsuitism (no affected areas, n = 2 [3.57%]; one affected area, n = 33 [58.93%]; two or more affected areas, n = 21 [37.50%]).

Intraclass correlation coefficients (*ICC*) (two-way mixed, single measures with absolute agreement definition) were computed to determine inter-rater reliability (please note that in our pre-registration it was stated that only one set of direct measurements was obtained: this was incorrect [two sets of direct measurements were available for a subsample of participants]). These revealed that inter-rater reliability was low for the direct measures: R2D:4D, *ICC* (n = 53) = 0.444, *p* < 0.001; L2D:4D, *ICC* (n = 52) = 0.614, *p* < 0.001; D_[R−L]_, *ICC* (n = 52) = 0.462, *p* < 0.001, but higher for the photocopy measures: R2D:4D, *ICC* (n = 70) = 0.829, *p* < 0.001; L2D:4D, *ICC* (n = 72) = 0.885, *p* < 0.001; D_[R−L]_, *ICC* (n = 66) = 0.811, *p* < 0.001. Contrary to expectation, the direct and photocopy measurements were uncorrelated: R2D:4D, *r*(65) = 0.065, *p* = 0.602; L2D:4D, *r*(66) = − 0.029, *p* = 0.814; D_[R−L]_, *r*(61) =  − 0.017, *p* = 0.893.

Descriptive statistics for digit ratio and maternal hormone variables are presented in Table 1. The hormonal measures did not differ in regard to fetal sex. L2D:4D measured from photocopies was lower in males than females, though all other comparisons were non-significant. Compared with direct measures, photocopies yielded lower R2D:4D (direct: *M* = 0.97, *SD* = 0.08; photocopy: *M* = 0.93, *SD* = 0.05), *t*(66) = 3.424, *p* = 0.001, *d* = 0.572, and L2D:4D (direct: *M* = 0.97, *SD* = 0.07; photocopy: *M* = 0.94, *SD* = 0.05), *t*(67) = 2.642, *p* = 0.010, *d* = 0.460. There was no difference for D_[R−L]_ (direct: *M* = 0.00, *SD* = 0.08; photocopy: *M* = – 0.01, *SD* = 0.06), *t*(62) = 1.068, *p* = 0.290, *d* = 0.192. Due to low reliability of the direct measures, and because they did not correlate with those obtained from photocopies, further analyses utilise photocopy measures only.

**Table 1:** Descriptive statistics for digit ratio (2D:4D) and hormone variables.

	Overall sample			Males			Females			Comparison			
	*n*	*M*	*SD*	*n*	*M*	*SD*	*n*	*M*	*SD*	*t*	*df*	*p*	*d*
Testosterone (nmol)	122	0.77	0.47	54	0.76	0.43	68	0.78	0.50	0.151	119.070	0.880	0.027
Estradiol^a^	122	0.97	0.42	54	1.01	0.44	68	0.93	0.41	− 0.947	108.810	0.346	− 0.174
T:E ratio^b^	122	0.09	0.05	54	0.08	0.05	68	0.09	0.05	0.634	115.350	0.528	0.115
R2D:4D (direct)	89	0.97	0.07	39	0.95	0.07	50	0.98	0.07	1.469	84.948	0.146	0.310
L2D:4D (direct)	88	0.97	0.07	39	0.97	0.08	49	0.96	0.06	− 0.584	72.137	0.561	− 0.128
D_[R−L]_ (direct)	88	0.00	0.09	39	− 0.02	0.07	49	0.01	0.09	1.633	85.973	0.106	0.342
R2D:4D (photocopy)	73	0.93	0.05	37	0.92	0.06	36	0.93	0.04	0.796	64.316	0.429	0.185
L2D:4D (photocopy)	74	0.94	0.05	35	0.93	0.05	39	0.95	0.04	**2.401**	**70.389**	**0.019**	**0.560**
D_[R−L]_ (photocopy)	69	− 0.01	0.07	33	0.00	0.08	36	− 0.02	0.05	− 1.294	55.454	0.201	− 0.317

Sample sizes differ for 2D:4D variables because measurements were only taken directly or indirectly from some participants; additionally, in some cases it was only possible to collect data for the right or left hand, e.g., because the second and/or fourth fingertips were missing from the photocopied images. Equal variances were not assumed for each of the independent samples t-tests reported here. Effect displayed in bold is statistically significant (*p* < 0.05, two-tailed).

^a^Estradiol was measured in nmol but the values reported here are divided by 10,000 for ease of interpretation.

^b^T:E was calculated as T (nmol)/E (nmol) but the values reported here are multiplied by 1000 for ease of interpretation.

**Figure 1:**
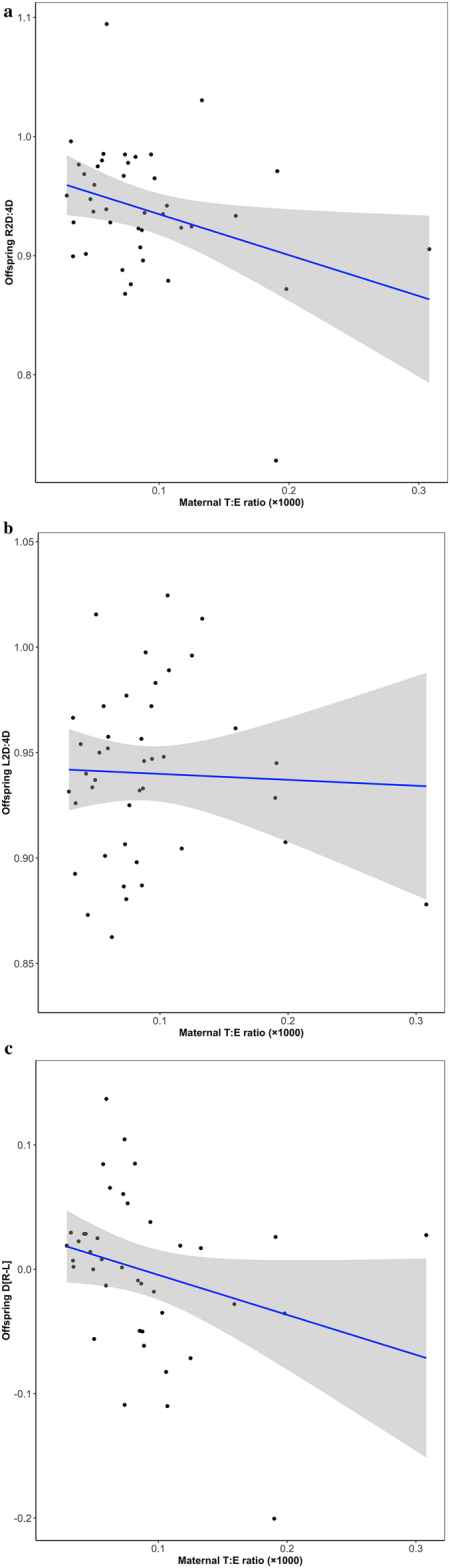
Scatterplots showing the associations between maternal T:E ratio and children’s (**a**) R2D:4D, (**b**) L2D:4D, and (**c**) D_[R−L]_. Raw data (not controlled for covariates) are shown; T:E ratio is multiplied by 1000.

Although not in our pre-registration plan, we report bivariate associations between maternal hormones and children’s digit ratios to facilitate comparison with studies that have not controlled for covariates (Table 2). T:E correlated negatively with R2D:4D and D_[R−L]_ but there was no association with L2D:4D (Fig. 1). None of the other hormone-digit ratio correlations were statistically significant. In our pre-registration, we specified that we would conduct bootstrapped (10,000 resamples) hierarchical multiple regression analyses (Step 1: enter covariates; Step 2: enter T, E, and the T × sex and E × sex interaction terms; Step 3: enter T:E ratio and the T:E × sex interaction term). However, in the interests of parsimony, we decided instead to run separate models for each predictor (along with its respective interaction term with sex). We included the following covariates: infant sex (0 = female, 1 = male), maternal PCOS status (1 = absent, 2 = present), maternal hirsutism (1 = no areas affected, 2 = one area affected, 3 = more than one area affected), infant’s birth weight (grams), infant’s age at follow-up corrected for gestational age (days) (we did not include child’s birth length as specified in our pre-registration because this variable was not made available). Since the covariates were included for the sole purpose of controlling for factors that may be associated with hormonal profiles during pregnancy and infant growth factors that could affect 2D:4D, we report in Table 2 the effect size estimates and bias corrected and accelerated 95% confidence intervals (BCa 95% CIs) for the predictors and not the covariates. Although T:E was no longer significantly associated with R2D:4D and D_[R−L]_, T correlated positively with L2D:4D and negatively with D_[R−L]_.

**Table 2:** Associations between maternal hormone concentrations and offspring digit ratios.

	R2D:4D			L2D:4D			D_[R−L]_		
	*n*	*ES*	*BCa 95% CI*	*n*	*ES*	*BCa 95% CI*	*n*	*ES*	*BCa 95% CI*
**Bivariate**									
Testosterone	41	0.023	− 0.252 to 0.265	42	0.190	− 0.114 to 0.465	40	− 0.115	− 0.437 to 0.177
Estradiol	41	0.305	− 0.039 to 0.516	42	0.050	− 0.290 to 0.390	40	0.236	− 0.144 to 0.486
T:E ratio	**41**	**− 0.337**	**− 0.631 to − 0.018**	42	− 0.038	− 0.412 to 0.288	**40**	**− 0.285**	**− 0.613 to − 0.014**
**Multivariate**									
Testosterone	37	− 0.022	− 0.069 to 0.035	**37**	**0.056**	**0.008 to 0.098**	**36**	**− 0.077**	**− 0.125 to − 0.013**
Testosterone × sex	37	0.042	− 0.081 to 0.233	37	− 0.051	− 0.137 to 0.028	36	0.086	− 0.062 to 0.236
Estradiol	37	0.027	− 0.039 to 0.079	37	0.033	− 0.032 to 0.099	36	− 0.006	− 0.091 to 0.062
Estradiol × sex	37	− 0.004	− 0.010 to 0.137	37	− 0.045	− 0.138 to 0.061	36	0.026	− 0.090 to 0.146
T:E ratio	37	− 0.458	− 0.795 to 0.111	37	0.130	− 0.418 to 0.726	36	− 0.562	− 1.205 to 0.073
T:E ratio × sex	37	0.360	− 0.511 to 1.007	37	− 0.258	− 1.002 to 0.752	36	0.600	− 0.481 to 1.413

BCa 95% CI = bias corrected and accelerated 95% confidence intervals.

Bivariate analyses are boostrapped (10,000 resamples) Pearson’s correlations; multivariate analyses are bootstrapped (10,000 resamples) multiple linear regression. ES = effect size (Pearson’s *r* for bivariate analyses; β for multivariate analyses). The following variables were included as covariates in the multivariate analyses: child’s sex (0 = female, 1 = male), maternal polycystic ovary syndrome (PCOS) status (1 = absent, 2 = present), maternal hirsutism score (1 = no areas affected, 2 = one area affected, 3 = more than one area affected), child’s birth weight (grams), child’s age at follow-up corrected for gestational age (days). Statistically significant effects (i.e., those for which the BCa 95% CIs do not include 0) are presented in bold.

## Discussion

The current study aimed to investigate whether maternal circulatory T:E ratio in early pregnancy predicts digit ratios in infancy. Our main prediction was that a high T:E ratio would correlate with low (male-typical) R2D:4D and L2D:4D, and that there would be no association with D_[R−L]_. Although we detected the predicted association with R2D:4D, T:E did not correlate with L2D:4D, and there was a negative correlation with D_[R−L]_. However, once covariates (including sex, and the relevant hormone × sex interaction term) were controlled for, T:E did not correlate significantly with any digit ratio variable, although T correlated positively with L2D:4D and negatively with D_[R−L]_. It is unclear why the results changed in this manner with the inclusion of covariates. However, we do not attempt to provide specific explanations for this, as they would necessarily be speculative in nature.

A negative correlation between maternal T:E ratio and offspring R2D:4D would be consistent with the theory that differential prenatal exposure to sex hormones affects development of digit ratios^1,2,4,6,7^, and in line with results from studies of human infants^3^ and experimental animal research^5^. However, this effect was not significant after controlling for covariates, and other research has reported no correlation between R2D:4D and the T:E ratio measured from amniotic fluid^41^ or between R2D:4D and the androgen-to-estrogen ratio measured from perinatal umbilical cord blood^50^. Additionally, the only other study to investigate offspring digit ratio in relation to maternal sex hormones in early pregnancy^60^ did not examine the T:E ratio. Further research using larger samples will be required to determine whether these variables are meaningfully related.

Contrary to our pre-registered prediction, a significant negative correlation between maternal T:E ratio and D_[R−L]_ was observed in bivariate association. However, this did not remain significant after controlling for demographic and obstetric covariates. This may therefore be considered consistent with observations that D_[R−L]_ does not correlate with sex hormones measured from amniotic fluid^40,41^, umbilical cord blood^48,50^ and second trimester maternal plasma^40^, and that it does not differ between people with and without CAH^10^. Although Baxter et al.^61^ recently reported a significant negative correlation between the urinary testosterone-to-estrone conjugate ratio of pregnant Titi monkeys and the D_[R−L]_ of their offspring, the effect reported there also did not retain statistical significance once covariates had been controlled for. It further remains unclear to what degree, if at all, maternal urinary sex hormone concentrations relate to those of the developing fetus.

The current study adds to a literature replete with inconsistent findings and replication failures^11^. In particular, there appears to be a concerning pattern in which smaller studies attempting to test the 2D:4D validity question report positive findings and larger ones do not. For example, small studies of CAH^1,62^, which have been heavily cited in the literature, have reported significant effects, whereas larger ones have not^63–65^. The same pattern is observed for twin research, with some early small studies observing significant effects^30,35^ but the largest in the area reporting only null findings^36^. Similarly, the negative correlation between amniotic T:E ratio and R2D:4D reported by Lutchmaya et al.^3^ was not replicated in a larger cohort^41^. The current study may also fit this general pattern: although some statistically significant effects were observed, the larger study by Barrett et al.^60^ found no correlation between early pregnancy maternal sex hormone concentrations and the 2D:4D of their children.

These issues present a serious challenge to the credibility of digit ratio research, particularly when considered in conjunction with the ease with which data can be collected and the considerable researcher degrees of freedom afforded at the analysis stage^66,67^. For instance, it has been noted that researchers often examine several digit ratio predictor variables in the same study (e.g., R2D:4D, L2D:4D, D_[R−L]_, and the average of R2D:4D and L2D:4D [M2D:4D]) and also stratify their analyses by sex^10,14,68^. Unless effective controls for alpha inflation are in place, this necessarily increases the chances of observing statistically significant effects, and, hence, making Type 1 errors. This problem is further compounded when researchers measure multiple outcome variables^68^, and particularly so if not all of those outcomes are reported^67,69^. A further issue is that one significant effect in the predicted direction may be taken as evidence in favour of rejecting the null hypothesis despite the greater weight of evidence being in favour of its acceptance^10^. To take a conservative example: if researchers were to examine R2D:4D and L2D:4D, separately in males and females, in relation to a single outcome variable, one statistically significant effect (e.g., for R2D:4D in males) might be emphasised over three concurrent null results for the same hypothesis (i.e., null effects for L2D:4D in males and for R2D:4D and L2D:4D in females). Such practices do not only lead to biased interpretations of individual datasets, but they also make it difficult to detect publication bias^10^, a problem which may be prevalent within the digit ratio literature^10,14,70^. This is because if there is a bias for publishing positive findings, it may be irrelevant in this field whether a statistically significant effect is found in relation to the right hand or left hand or in males or in females etc. An obvious way to address this issue going forwards is to pre-register studies with specific a priori hypotheses, predictions, and analysis plans, and to note in publications where analyses have deviated from these^14,71,72^.

R2D:4D and L2D:4D values in the current study were lower when measured from photocopies than when measured directly from participants’ hands. This corroborates findings from studies of older populations^73–75^, and implies that the process of photocopying may distort the soft tissue in a way that acts differentially across the second and fourth fingers. What was more surprising was that the direct and photocopy measures were uncorrelated in our sample, as previous research has identified both techniques to be reliable^76,77^. As (after removal of outliers) 2D:4D measurements taken directly (self-measured) from young adults have been reported to correlate moderately (R2D:4D, *r* = 0.518; L2D:4D, *r* = 0.409) with those taken from photocopies (researcher-measured)^78^, the lack of intercorrelation observed within the current study may simply reflect the difficulty associated with obtaining accurate measurements directly from the hands of toddlers. Correlations between direct and photocopy measures of digit ratio in young children may generally be fairly low (e.g., R2D:4D: *r* = 0.421; L2D:4D: *r* = 0.373 [Constantinescu, 2009, p. 32^65^]) but it remains unclear why we observed no association at all. We suggest that, despite the slight distortion caused, photocopies/scans may be the most effective method of obtaining reliable data in this population. Although one might still make the case for using direct measurements, we examined those derived from photocopies because they are more likely to be reliable. This is indicated by the higher *ICC*s and lower *SD*s observed in the current study, and by previous research reporting computer-assisted measurements to yield the most reliable results^76,77^. Additionally, we used these measures because it would be possible to check the data against the original photocopies whereas this is not so for the direct measurements. Analysis of both sets of measurements would have increased the number of null hypothesis significance tests used, and, therefore, the chances of making Type 1 errors.

The current study has strength in that it not only reported on two hormones in isolation and their interactions with fetal sex, but also examined the T:E ratio, a variable posited to play a key role in the determination of digit ratios^3–6^. It is additionally important that sex hormones were assayed in close proximity to the time at which they are hypothesised to exert their greatest influence on digit ratio development^59^. However, some limitations should also be considered. Though comparable with previous research^3,41,48,50^, our sample is only modest in size; it therefore, lacks the statistical power required to detect small effects. Second, although inter-rater reliability for digit ratios measured from photocopies was high, it is notable that these measures did not correlate with those taken directly from participants’ hands. Third, fetal steroids were not measured directly, and it remains unclear how representative maternal serum samples may be of the fetal circulation. Previous research has shown estradiol measured from second trimester amniotic fluid to be positively correlated with that present in maternal serum sampled in the second and third trimesters^42^, and human chorionic gonadotropin (hCG) levels measured from amniotic fluid and maternal serum are positively correlated during the second (but not third) trimester^79^. Conversely, although there are exceptions (e.g.^80,81^), most studies have found maternal serum testosterone to be unrelated to fetal sex^42,44,82–85^, and testosterone concentrations in the maternal and fetal plasma have been reported to be uncorrelated^43^. Considering that digit ratios have been hypothesised to relate primarily to sex steroid concentrations present within the fetal rather than maternal circulation, the findings of the present study should therefore be interpreted cautiously.

## Conclusions

The current study contributes the first pre-registered analysis of 2D:4D in relation to maternal sex hormone concentrations, as well as the first empirical test of whether the right-left difference in digit ratios (D_[R−L]_) is correlated with hormonal concentrations measured during early pregnancy. We attempted to test the hypothesis that mothers’ T:E ratio is a predictor of their offspring’s digit ratios. Although we observed statistically significant effects linking high T:E with low R2D:4D and D_[R−L]_ at 18–22-month follow-up, neither effect remained statistically significant once covariates had been controlled for. Furthermore, multivariate analyses revealed that T correlated positively with L2D:4D and negatively with D_[R−L]_, the first of these effects being inconsistent with well established theory. Taken together, the results of this study suggest that further research with larger sample sizes will be required to determine whether digit ratios are valid proxy measures of maternal sex hormone exposure.

## Methods

### Participants

Mothers (n = 217) were recruited early in their pregnancy, during or before their routine 20-week ultrasound scan, as part of the Cambridge Ultrasound Siblings and Parents Study (CUSP) at the Rosie Hospital, Cambridge University Hospitals NHS Foundation Trust. Ethical approval was provided by the East of England Cambridge Central Research Ethics Committee (ref: 16/EE/0004) and the Research and Development department of Cambridge University Hospitals. All mothers gave written informed consent for access to their pregnancy-related clinical records, test results, and biological samples obtained during routine clinical care, and the procedures were conducted in accordance with the Declaration of Helsinki. Inclusion criteria were as follows: (1) little/no consumption of alcohol during pregnancy, (2) no smoking or recreational drug use during pregnancy, (3) a singleton fetus whose measurements did not indicate intrauterine growth restriction or large-for-gestational age, (4) absence of any major fetal anomalies, and (5) birth of a clinically healthy baby. At time of scan mothers were asked to complete a pregnancy history questionnaire to self-report metabolic, reproductive, and diagnosed conditions. Detailed description of this sample has already been reported elsewhere^46,86^.

### Hormone assays

Serum samples were collected by a specialist phlebotomist at the Rosie Hospital and stored at − 80 °C, as part of a national screening programme at the end of first trimester/start of second trimester (*M* = 12.7 [*SD* = 0.7] weeks gestation^46^) for biomarkers of Down’s Syndrome and other conditions. Samples from CUSP participants (n = 122) were thawed and transferred to separate vials (1 ml aliquots per sample), which were anonymised and sent for analysis at the Core Biochemical Assays Laboratory (CBAL) at Addenbrookes Hospital, Cambridge.

Concentrations of testosterone (T), estradiol (E), dehydroepiandrosterone sulphate (DHEAS), progesterone (P), and sex hormone-binding globulin (SHBG) were measured. Samples were analysed on a DiaSorin Liaison^®^ XL automated immunoassay analyser using a one-step competitive chemiluminescence immunoassay for each hormone and two monoclonal antibodies for each peptide. All reagents, standards and consumables are those supplied by DiaSorin (DiaSorin S.p.A, 13040 Saluggia [VC], Italy). Although SHBG was assayed, allowing for estimation of the free testosterone index and the free estradiol index, we instead include the total hormone levels in our statistical models. This is because SHBG does not easily cross the placenta^87^, and so it is unclear whether SHBG in the maternal serum is reflective of fetal bioactivity.

### Digit ratio (2D:4D) measurements

Parents and infants were invited for an in-person follow-up visit to measure 2D:4D and physical growth (infants’ *M*_age_ = 19.87, *SD* = 0.86, range = 18.20–21.95 months). Direct measures of finger length were taken by Research Assistants using a standard tape measure. A Canon LiDE 300 flatbed scanner was used to scan infants’ left and right hands, and colour images were made at a resolution of 2400 × 4800 dpi. 2D:4D ratios were calculated from these by two researchers (GR and EA) using AutoMetric 2.2 for Windows^88^.

### Statistical analysis

We computed the averages for R2D:4D, L2D:4D, and D_[R−L]_ across the two sets of measurements (separately for direct and photocopy measures). We checked for correlation (Pearson’s tests) and differences (paired samples *t*-tests) between the direct and photocopy measures, and used independent samples *t*-tests to examine for sex differences. We then used bootstrapped (10,000 resamples) Pearson’s correlations and multiple linear regression analyses to determine whether the maternal hormones (T, E, and T:E ratio) were associated with digit ratio (R2D:4D, L2D:4D, and D_[R−L]_). We included the following covariates: child’s sex (0 = female, 1 = male), maternal polycystic ovary syndrome (PCOS) status (1 = absent, 2 = present), maternal hirsutism score (1 = no areas affected, 2 = one area affected, 3 = more than one area affected), child’s birth weight (grams), child’s age at follow-up corrected for gestational age (days). We included covariates to control for factors related to the maternal hormonal environment (PCOS and hirsuitism are associated with elevated androgen concentrations^89,90^) and infant factors related to growth trajectories that could affect 2D:4D (2D:4D may fluctuate considerably during early postnatal life^91,92^; it may also correlate with birth weight, although empirical findings are mixed^93–96^). We also included infant sex as a covariate because 2D:4D exhibits marked sex differences^9^ and because associations with prenatal hormonal variables could differ between males and females^39^.

When utilising a bootstrapping approach, a specified number of resamples (in this case, 10,000) the size of the original is drawn with replacement from the available data. The chosen statistic is then computed for each resample. These resamples are considered equivalent to samples derived in the usual way from an infinitely large population with similar characteristics to those of the observed data. The variation among resamples indicates what would be expected from sampling variation under such circumstances (see Loehlin et al., p. 300–301^97^). We used bootstrapping because it does not assume a normal distribution of the error term^98^, and may be advantageous when examining variables that exhibit marked deviations from the normal distribution as well as presence of datapoints that would be considered outliers in the context of a normal distribution^41,97,99,100^.

## Data Availability

The datasets generated and/or analysed during the current study are not publicly available due to limited ethics approval for the wider clinical study (CUSP) by CUH and to the specific consent provided by the participants. They may be available from the corresponding author on reasonable request and pending approval of any future analyses by CUH. The R script used to run the analysis is available on the Open Science Framework: https://osf.io/uj3mv/.

## References

[1] Brown WM, Hines M, Fane BA, Breedlove SM (2002). Masculinized finger length patterns in human males and females with congenital adrenal hyperplasia. Horm. Behav..

[2] Manning JT, Scutt D, Wilson J, Lewis-Jones DI (1998). The ratio of 2nd to 4th digit length: A predictor of sperm numbers and concentrations of testosterone, luteinizing hormone and oestrogen. Hum. Reprod..

[3] Lutchmaya S, Baron-Cohen S, Raggatt P, Knickmeyer RC, Manning JT (2004). 2nd to 4th digit ratios, fetal testosterone and estradiol. Early Human Dev..

[4] Manning JT (2011). Resolving the role of prenatal sex steroids in the development of digit ratio. Proc. Natl. Acad. Sci..

[5] Zheng Z, Cohn MJ (2011). Developmental basis of sexually dimorphic digit ratios. Proc. Natl. Acad. Sci. USA.

[6] Manning JT (2002). Digit Ratio: A Pointer to Fertility, Behavior, and Health.

[7] Manning JT, Kilduff L, Cook C, Crewther B, Fink B (2014). Digit ratio (2D:4D): A biomarker for prenatal sex steroids and adult sex steroids in challenge situations. Front. Endocrinol..

[8] Manning JT, Fink B, Mason L, Kasielska-Trojan A, Trivers R (2022). The effects of sex, nation, ethnicity, age and self-reported pubertal development on participant-measured right-left 2D:4D (Dr-l) in the BBC internet study. J. Biosoc. Sci..

[9] Hönekopp J, Watson S (2010). Meta-analysis of digit ratio 2D:4D shows greater sex difference in the right hand. Am. J. Hum. Biol..

[10] Richards G (2020). Digit ratio (2D:4D) and congenital adrenal hyperplasia (CAH): Systematic literature review and meta-analysis. Horm. Behav..

[11] Richards G (2017). What is the evidence for a link between digit ratio (2D:4D) and direct measures of prenatal sex hormones?. Early Human Dev..

[12] Fusar-Poli L (2021). Second-to-fourth digit ratio (2D:4D) in psychiatric disorders: A systematic review of case-control studies. Clin. Psychopharmacol. Neurosci..

[13] Manning JT, Reimers S, Baron-Cohen S, Wheelwright S, Fink B (2010). Sexually dimorphic traits (digit ratio, body height, systemizing-empathizing scores) and gender segregation between occupations: Evidence from the BBC internet study. Personality Individ. Differ..

[14] Neyse L, Johannesson M, Dreber A (2021). 2D:4D does not predict economic preferences: Evidence from a large, representative sample. J. Econ. Behav. Organ..

[15] de Fonseca CAD (2022). Digital biomarker 2D:4D as a predictor of cancer: A systematic review. Early Human Dev..

[16] Pratt TC, Turanovic JJ, Cullen FT (2016). Revisiting the criminological consequences of exposure to fetal testosterone: A meta-analysis of the 2D:4D digit ratio. Criminology.

[17] Hönekopp J, Schuster M (2010). A meta-analysis on 2D:4D and athletic prowess: Substantial relationships but neither hand out-predicts the other. Personality Individ. Differ..

[18] Pasanen BE (2022). The relationship between digit ratio (2D:4D) and muscular fitness: A systematic review and meta-analysis. Am. J. Hum. Biol..

[19] Berenbaum SA, Bryk KK, Nowak N, Quigley CA, Moffat S (2009). Fingers as a marker of prenatal androgen exposure. Endocrinology.

[20] van Hemmen J, Cohen-Kettenis PT, Steensma TD, Veltman DJ, Bakker J (2017). Do sex differences in CEOAEs and 2D:4D ratios reflect androgen exposure? A study in women with complete androgen insensitivity syndrome. Biol. Sex Differ..

[21] Wallen K (2009). Does finger fat produce sex differences in second to fourth digit ratios?. Endocrinology.

[22] Zitzmann M, Nieschlag E (2003). The CAG repeat polymorphism within the androgen receptor gene and maleness. Int. J. Androl..

[23] Manning JT, Bundred PE, Newton DJ, Flanagan BF (2003). The second to fourth digit ratio and variation in the androgen receptor gene. Evol. Hum. Behav..

[24] Voracek M (2014). No effects of androgen receptor gene CAG and GGC repeat polymorphisms on digit ratio (2D:4D): A comprehensive meta-analysis and critical evaluation of research. Evol. Hum. Behav..

[25] Hönekopp, J. No evidence that 2D:4D is related to the number of CAG repeats in the androgen receptor gene. *Front. Endocrinol*. **4**, (2013). 10.3389/fendo.2013.00185 PMC3851970 24367354

[26] Zhang K (2020). Revisiting the relationships of 2D:4D with androgen receptor (AR) gene and current testosterone levels: Replication study and meta-analyses. J. Neurosci. Res..

[27] Manning JT, Kilduff LP, Trivers R (2013). Digit ratio (2D:4D) in Klinefelter’s syndrome. Andrology.

[28] Chang S (2015). Anthropometry in Klinefelter syndrome—Multifactorial influences due to CAG length, testosterone treatment and possibly intrauterine hypogonadism. J. Clin. Endocrinol. Metab..

[29] Ratcliffe SG (1994). Prenatal testosterone levels in XXY and XYY males. Horm. Res..

[30] Voracek M, Dressler SG (2007). Digit ratio (2D:4D) in twins: Heritability estimates and evidence for a masculinized trait expression in women from opposite-sex pairs. Psychol. Rep..

[31] Gobrogge KL, Breedlove SM, Klump KL (2008). Genetic and environmental influences on 2D:4D finger length ratios: A study of monozygotic and dizygotic male and female twins. Arch. Sex. Behav..

[32] Medland SE, Loehlin JC (2008). Multivariate genetic analyses of the 2D:4D ratio: Examining the effects of hand and measurement technique in data from 757 twin families. Twin Res. Hum. Genet..

[33] Paul SN, Kato BS, Cherkas LF, Andrew T, Spector TD (2006). Heritability of the second to fourth digit ratio (2d:4d): A twin study. Twin Res. Hum. Genet..

[34] Ahrenfeldt LJ, Christensen K, Segal NL, Hur Y-M (2020). Opposite-sex and same-sex twin studies of physiological, cognitive and behavioral traits. Neurosci. Biobehav. Rev..

[35] van Anders SM, Vernon PA, Wilbur CJ (2006). Finger-length ratios show evidence of prenatal hormone-transfer between opposite-sex twins. Horm. Behav..

[36] Medland SE, Loehlin JC, Martin NG (2008). No effects of prenatal hormone transfer on digit ratio in a large sample of same- and opposite-sex dizygotic twins. Personality Individ. Differ..

[37] Hiraishi K, Sasaki S, Shikishima C, Ando J (2012). The second to fourth digit ratio (2D:4D) in a Japanese twin sample: Heritability, prenatal hormone transfer, and association with sexual orientation. Arch. Sex. Behav..

[38] Cohen-Bendahan C (2005). Biological Roots of Sex Differences: A Longitudinal Twin Study.

[39] Ventura T, Gomes MC, Pita A, Neto MT, Taylor A (2013). Digit ratio (2D:4D) in newborns: Influences of prenatal testosterone and maternal environment. Early Human Dev..

[40] Richards G, Gomes M, Ventura T (2019). Testosterone measured from amniotic fluid and maternal plasma shows no significant association with directional asymmetry in newborn digit ratio (2D:4D). J. Dev. Orig. Health Dis..

[41] Richards G, Browne W, Constantinescu M (2020). Digit ratio (2D:4D) and amniotic testosterone and estradiol: An attempted replication of Lutchmaya et al. (2004). J. Developmental Origins Health Disease..

[42] van de Beek C, Thijssen JHH, Cohen-Kettenis PT, van Goozen SHM, Buitelaar JK (2004). Relationships between sex hormones assessed in amniotic fluid, and maternal and umbilical cord serum: What is the best source of information to investigate the effects of fetal hormonal exposure?. Horm. Behav..

[43] Rodeck CH, Gill D, Rosenberg DA, Collins WP (1985). Testosterone levels in midtrimester maternal and fetal plasma and amniotic fluid. Prenat. Diagn..

[44] Hines M (2002). Testosterone during pregnancy and gender role behavior of preschool children: A longitudinal, population study. Child Dev..

[45] Udry JR, Morris NM, Kovenock J (1995). Androgen effects on women’s gendered behaviour. J. Biosoc. Sci..

[46] Tsompanidis A (2021). Maternal steroid levels and the autistic traits of the mother and infant. Mol. Autism.

[47] Barona M, Kothari R, Skuse D, Micali N (2015). Social communication and emotion difficulties and second to fourth digit ratio in a large community-based sample. Mol. Autism.

[48] Hickey M (2010). Maternal and umbilical cord androgen concentrations do not predict digit ratio (2D:4D) in girls: A prospective cohort study. Psychoneuroendocrinology.

[49] Harris JA, Vernon PA, Boomsma DI (1998). The heritability of testosterone: A study of dutch adolescent twins and their parents. Behav. Genet..

[50] Hollier LP (2015). Adult digit ratio (2D:4D) is not related to umbilical cord androgen or estrogen concentrations, their ratios or net bioactivity. Early Hum. Dev..

[51] Whitehouse AJO (2015). Prenatal testosterone exposure is related to sexually dimorphic facial morphology in adulthood. Proc. R. Soc. B Biol. Sci..

[52] Çetin R, Can M, Özcan E (2016). The relationship between testosterone and oestrogen level of the cord blood and length of fingers of newborns 2D:4D. Balıkesır Health Sci. J..

[53] Mitsui T (2015). Effects of prenatal Leydig cell function on the ratio of the second to fourth digit lengths in school-aged children. PLoS ONE.

[54] Mitsui T (2016). Effects of adrenal androgens during the prenatal period on the second to fourth digit ratio in school-aged children. Steroids.

[55] van Leeuwen B (2020). Do sex hormones at birth predict later-life economic preferences? Evidence from a pregnancy birth cohort study. Proc. R. Soc. B Biol. Sci..

[56] Hollier LP, Keelan JA, Hickey M, Maybery MT, Whitehouse AJO (2014). Measurement of androgen and estrogen concentrations in cord blood: Accuracy, biological interpretation, and applications to understanding human behavioral development. Front. Endocrinol..

[57] Galis F, ten Broek CMA, van Dongen S, Wijnaendts LCD (2010). Sexual dimorphism in the prenatal digit ratio (2D:4D). Arch. Sex. Behav..

[58] Malas MA, Dogan S, Evcil EH, Desdicioglu K (2006). Fetal development of the hand, digits and digit ratio (2D:4D). Early Hum. Dev..

[59] Manning JT, Fink B (2017). Are there any “direct” human studies of digit ratio (2D:4D) and measures of prenatal sex hormones?. Early Hum. Dev..

[60] Barrett E (2020). Digit ratio, a proposed marker of the prenatal hormone environment, is not associated with prenatal sex steroids, anogenital distance, or gender-typed play behavior in preschool age children. J. Dev. Orig. Health Dis..

[61] Baxter A, Wood EK, Witczak LR, Bales KL, Higley JD (2019). Sexual dimorphism in titi monkeys’ digit (2D:4D) ratio is associated with maternal urinary sex hormones during pregnancy. Dev. Psychobiol..

[62] Ökten A, Kalyoncu M, Yariş N (2002). The ratio of second- and fourth-digit lengths and congenital adrenal hyperplasia due to 21-hydroxylase deficiency. Early Hum. Dev..

[63] Buck JJ, Williams RM, Hughes IA, Acerini CL (2003). In-utero androgen exposure and 2nd to 4th digit length ratio—Comparisons between healthy controls and females with classical congenital adrenal hyperplasia. Hum. Reprod..

[64] Nave G (2021). No evidence for a difference in 2D:4D ratio between youth with elevated prenatal androgen exposure due to congenital adrenal hyperplasia and controls. Horm. Behav..

[65] Constantinescu M (2009). Are Finger Ratios a Useful Measure of Androgenic Influences on Sexual Differentiation?.

[66] Gelman, A. & Loken, E. The garden of forking paths: Why multiple comparisons can be a problem, even when there is no “fishing expedition” or “p-hacking” and the research hypothesis was posited ahead of time. http://www.stat.columbia.edu/~gelman/research/unpublished/p_hacking.pdf (2013).

[67] Simmons JP, Nelson LD, Simonsohn U (2011). False-positive psychology: Undisclosed flexibility in data collection and analysis allows presenting anything as significant. Psychol. Sci..

[68] Putz DA, Gaulin SJC, Sporter RJ, McBurney DH (2004). Sex hormones and finger length: What does 2D:4D indicate?. Evol. Hum. Behav..

[69] Goldacre B (2008). Bad Science.

[70] Voracek M, Stieger S (2009). Replicated nil associations of digit ratio (2D:4D) and absolute finger lengths with implicit and explicit measures of aggression. Psicothema.

[71] Osu T (2021). Fluctuating asymmetry of finger lengths, digit ratio (2D:4D), and tattoos: A pre-registered replication and extension of Koziel et al. (2010). Early Hum. Development..

[72] Fossen FM, Neyse L, Johannesson M, Dreber A (2022). 2D:4D and self-employment: A preregistered replication study in a large general population sample. Entrepreneurship Theory Pract..

[73] Ribeiro E, Neave N, Morais RN, Manning JT (2016). Direct versus indirect measurement of digit ratio (2D:4D): A critical review of the literature and new data. Evol. Psychol..

[74] Fink B, Manning JT (2018). Direct versus indirect measurement of digit ratio: New data from Austria and a critical consideration of clarity of report in 2D:4D studies. Early Human Dev..

[75] Manning JT, Fink B, Neave N, Caswell N (2005). Photocopies yield lower digit ratios (2D:4D) than direct finger measurements. Arch. Sex. Behav..

[76] Kemper CJ, Schwerdtfeger A (2009). Comparing indirect methods of digit ratio (2D:4D) measurement. Am. J. Hum. Biol..

[77] Allaway HC, Bloski TG, Pierson RA, Lujan ME (2009). Digit ratios (2D:4D) determined by computer-assisted analysis are more reliable than those using physical measurements, photocopies, and printed scans. Am. J. Hum. Biol..

[78] Caswell N, Manning JT (2009). A comparison of finger 2D:4D by self-report direct measurement and experimenter measurement from photocopy: Methodological issues. Arch. Sex. Behav..

[79] Steier JA, Myking OL, Bergsjø PB (1999). Correlation between fetal sex and human chorionic gonadotropin in peripheral maternal blood and amniotic fluid in second and third trimester normal pregnancies. Acta Obstet. Gynecol. Scand..

[80] Meulenberg PMM, Hofman JA (1991). Maternal testosterone and fetal sex. J. Steroid Biochem. Mol. Biol..

[81] Klinga K, Bek E, Runnebaum B (1978). Maternal peripheral testosterone levels during the first half of pregnancy. Am. J. Obstet. Gynecol..

[82] Vlková B (2010). Testosterone and estradiol in maternal plasma and their relation to fetal sex. Prenat. Diagn..

[83] Nabi G, Aziz T, Amin M, Khan AA (2014). Effect of fetal sex on total levels of maternal serum testosterone. J. Biol. Life Sci..

[84] Firestein MR (2022). Elevated prenatal maternal sex hormones, but not placental aromatase, are associated with child neurodevelopment. Horm. Behav..

[85] Sarkar P, Bergman K, Fisk NM, O’Connor TG, Glover V (2007). Amniotic fluid testosterone: Relationship with cortisol and gestational age. Clin. Endocrinol..

[86] Aydin E (2020). Fetal Biometry and Early Behavioural Development.

[87] Hogeveen KN (2002). Human sex hormone–Binding globulin variants associated with hyperandrogenism and ovarian dysfunction. J. Clin. Investig..

[88] DeBruine, L. AutoMetric software for measurement of 2D:4D ratios (2006).

[89] Legro RS (2010). Total testosterone assays in women with polycystic ovary syndrome: Precision and correlation with hirsutism. J. Clin. Endocrinol. Metab..

[90] Al Kindi MK, Al Essry FS, Al Essry FS, Mula-Abed W-AS (2012). Validity of serum testosterone, free androgen index, and calculated free testosterone in women with suspected hyperandrogenism. Oman Med. J..

[91] Ernsten L, Körner LM, Heil M, Richards G, Schaal NK (2021). Investigating the reliability and sex differences of digit lengths, ratios, and hand measures in infants. Sci. Rep..

[92] Knickmeyer RC, Woolson S, Hamer RM, Konneker T, Gilmore JH (2011). 2D:4D ratios in the first 2 years of life: Stability and relation to testosterone exposure and sensitivity. Horm. Behav..

[93] Danborno B, Adebisi SS, Adelaiye AB, Ojo SA (2010). Relationship between digit ratio (2D:4D) and birth weight in Nigerians. Anthropologist.

[94] Kobus M, Sitek A, Rosset I, Pruszkowska-Przybylska P, Żądzińska E (2021). Association of prenatal sex steroid exposure estimated by the digit ratio (2D:4D) with birth weight, BMI and muscle strength in 6- to 13-year-old Polish children. PLoS ONE.

[95] McIntyre MH, Cohn BA, Ellison PT (2006). Sex dimorphism in digital formulae of children. Am. J. Phys. Anthropol..

[96] Ronalds G, Phillips DIW, Godfrey KM, Manning JT (2002). The ratio of second to fourth digit lengths: A marker of impaired fetal growth?. Early Human Dev..

[97] Loehlin JC, Medland SE, Martin NG (2009). Relative finger lengths, sex differences, and psychological traits. Arch. Sex. Behav..

[98] Diaconis P, Efron B (1983). Computer-intensive methods in statistics. Sci. Am..

[99] Richards G (2021). An examination of the influence of prenatal sex hormones on handedness: Literature review and amniotic fluid data. Horm. Behav..

[100] Wilke M, Schmithorst VJ (2006). A combined bootstrap/histogram analysis approach for computing a lateralization index from neuroimaging data. Neuroimage.

